# Nivolumab and Ipilimumab Combination Immunotherapy for Patients with Metastatic Collecting Duct Carcinoma

**DOI:** 10.1155/2021/9936330

**Published:** 2021-07-02

**Authors:** Tetsuya Danno, Shohei Iwata, Fusako Niimi, Sachi Honda, Haruka Okada, Takeshi Azuma

**Affiliations:** ^1^Department of Urology, Tokyo Metropolitan Tama Medical Center, 2-8-29 Musashidai, Fuchu, Tokyo 183-0042, Japan; ^2^Department of Pathology, Tokyo Metropolitan Tama Medical Center, 2-8-29 Musashidai, Fuchu, Tokyo 183-0042, Japan

## Abstract

Collecting duct carcinoma (CDC) is a rare, extremely aggressive form of renal cancer. Recently, immune checkpoint inhibitors (ICI), anti-programmed death-1 (PD-1) antibody, and anti-cytotoxic T lymphocyte-associated antigen 4 (CTLA-4) antibody were approved for use against metastatic renal cell carcinoma. We herein described two cases of metastatic renal collecting duct carcinoma treated with a combination immunotherapy consisting of nivolumab and ipilimumab. In the first case, which included a bone metastasis, the best response achieved was stable disease (SD) for one year. In the second case, which was accompanied by a lung metastasis, the best response achieved was a partial response. The outcome of these cases suggested that the combination of nivolumab and ipilimumab is effective against renal collecting duct carcinoma.

## 1. Introduction

Collecting duct carcinoma (CDC) is a rare form of renal cancer constituting fewer than 1% of renal cell carcinoma cases. CDC is extremely aggressive, with 40% of patients presenting a metastasis at the initial diagnosis. Because an effective, systemic therapy for metastatic CDC has not yet been established, the prognosis is poorer than that of clear cell carcinoma [1].

Azuma et al. published a prospective study of systemic therapy for CDC using a cisplatin-based regimen. However, the clinical benefits were limited, and the median survival time was less than 12 months. Targeted therapies have also had limited effect [1].

Recently, immune checkpoint inhibitors (ICI), anti-programmed death-1 (PD-1) antibody, and anti-cytotoxic T lymphocyte-associated antigen 4 (CTLA-4) antibody were approved as treatments for metastatic renal cell carcinoma (RCC). PD-1 and programmed death-1 ligand-1 (PD-L1) are expressed on T cells and cancer cells, respectively. Their interaction sends an inhibitory signal to T cells via PD-1 [2]. Cytotoxic T lymphocyte-associated antigen 4 (CTLA-4) is also expressed on CTLs and delivers an inhibitory signal to CTLs [3]. Using anti-PD-1 antibody or anti-CTLA-4 antibodies to block the interaction with their ligands can activate T cells against cancer cells.

Immunotherapy combining the anti-PD-1 and CTLA-4 antibodies has also been approved for the treatment of metastatic RCC. The anti-PD-1/anti-CTLA-4 antibody combination immunotherapy was more effective than anti-PD-1 antibody monotherapy. However, the efficacy of immunotherapies using ICI against CDC is unclear. We herein reported two cases which demonstrated the efficacy of the anti-PD-1/anti-CTLA-4 antibody combination immunotherapy against metastatic CDC.

## 2. Case Report

### 2.1. Case 1

**Figure 1:**
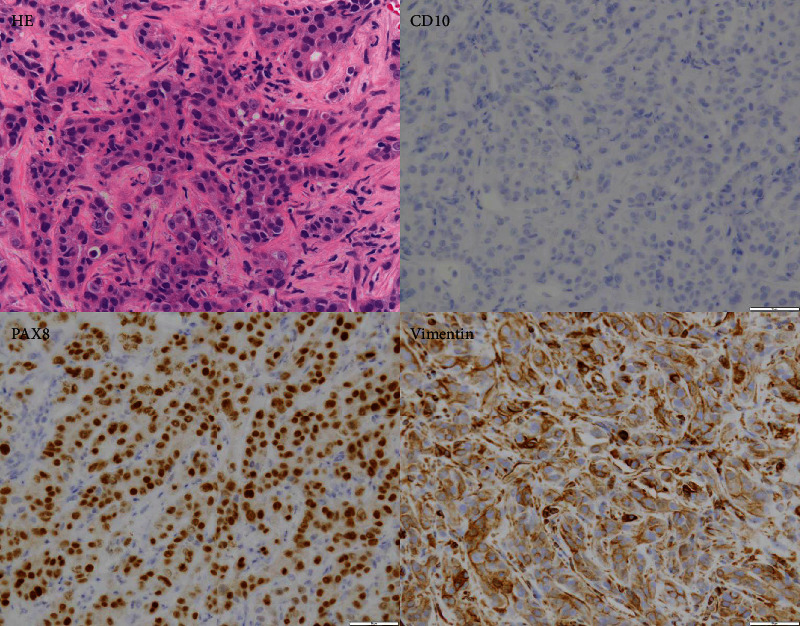
Hematoxylin and eosin staining and immunohistochemical staining of tissue from the renal biopsy in Case 1 (×400).

**Figure 2:**
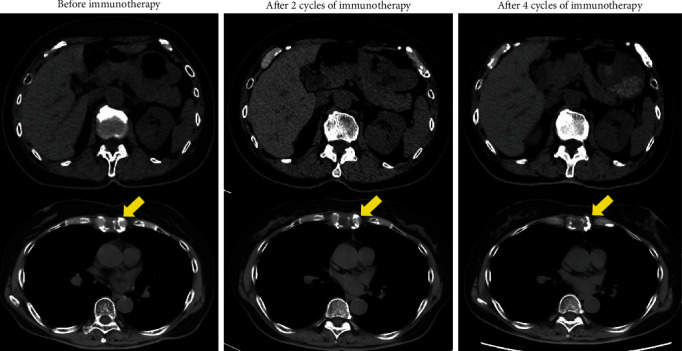
Computed tomography (CT) findings in Case 1. Abdominal CT showed no change in the right primary renal tumor or bone metastasis after four cycles of immunotherapy.

A 75-year-old female patient with a left renal mass and bone metastases was referred to our institution. A biopsy of the left renal mass was performed, and pathological analysis revealed collecting duct carcinoma (CDC) (PAX8+, Vimentin+, and CD10-) (Figure 1) [4]. Based on the International Metastatic Renal Cell Carcinoma Database Consortium (IMDC) risk score, the prognostic risk was determined to be intermediate (<1 year since the diagnosis). Nivolumab and ipilimumab were administered four times every three weeks; then, nivolumab monotherapy was administered every two weeks for maintenance. After completion of two cycles of the combination immunotherapy, computed tomography (CT) revealed a slight enlargement of the primary tumor and bone metastases but showed no change during the two additional cycles of the combination therapy or the nivolumab monotherapy (Figure 2). The best response achieved was stable disease (SD) lasting 23 months.

### 2.2. Case 2

**Figure 3:**
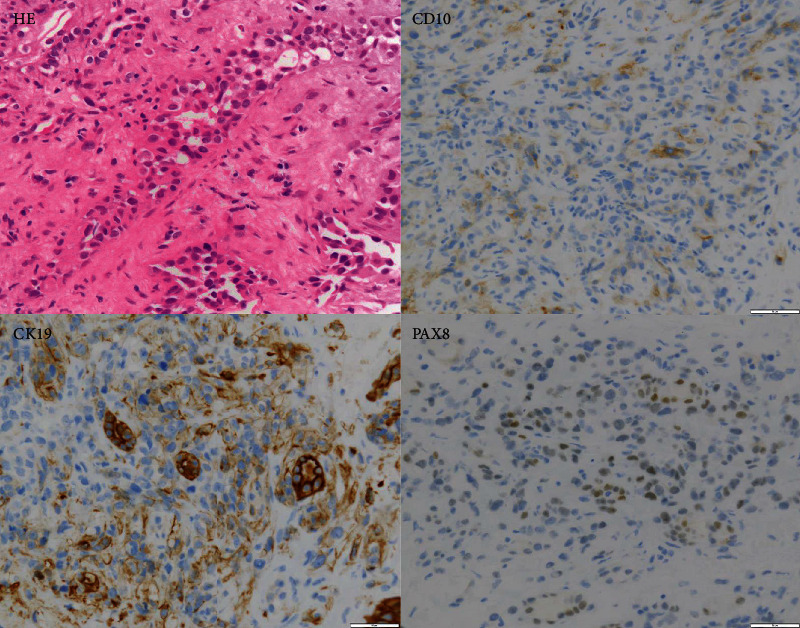
Hematoxylin and eosin staining and immunohistochemical staining of tissue from the renal biopsy in Case 2 (×400).

**Figure 4:**
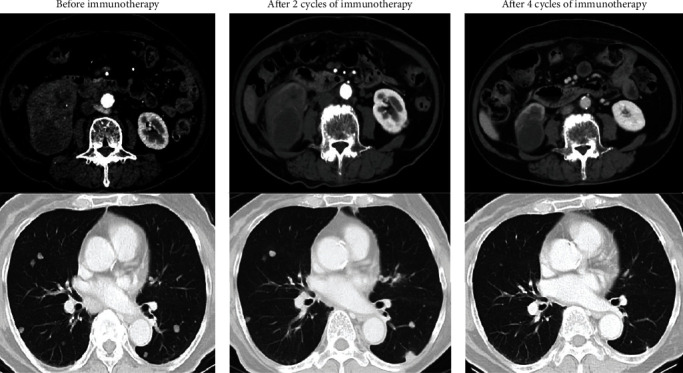
Computed tomography (CT) findings in Case 2 showing new lesions in the lung after two cycles of immunotherapy, which shrank following the two additional cycles.

A 79-year-old female patient had a right renal tumor and multiple metastases to the lungs and lymph nodes. Pathological analysis of a biopsy specimen of the right renal mass revealed CDC (CK19+, PAX8+, and CD10-) (Figure 3) [4]. Based on the International Metastatic Renal Cell Carcinoma Database Consortium (IMDC) risk score, her risk level was determined to be poor (neutrophilia, anemia, and duration < 1 year after diagnosis). Combination immunotherapy with nivolumab and ipilimumab was administered, and after two cycles, computed tomography (CT) revealed new bilateral lung lesions. After two additional cycles, all the tumors shrank markedly (Figure 4). However, the immunotherapy was unable to be continued due to rheumatoid arthritis development, an adverse event associated with the therapy. The best response achieved was a partial response (PR). The patient was followed up without treatment after four cycles of the combination therapy. After eight months, nivolumab was resumed because the lung metastases showed slight growth. Thereafter, SD continued for six months, indicating that the immunotherapy was able to suppress progression for 17 months.

## 3. Discussion

To the best of our knowledge, ICI was shown to be effective in treating five, previously reported cases of metastatic CDC. Four previous reports described the efficacy of nivolumab monotherapy, and only one study reported using the anti-PD-1 and CTLA-4 antibody combination immunotherapy [5–8]. The present study is the second report of the use of a combination immunotherapy against metastatic CDC.

In previous studies, two of five CDC cases were recurrences following a nephrectomy; one patient required a nephrectomy to reduce tumor volume. In the present study, the combination immunotherapy was promptly performed in both patients without performing a nephrectomy. In Case 2, there was no time for a nephrectomy due to the extremely swift progression of the cancer from the time of diagnosis. The previous reports demonstrated effectiveness of immunotherapy after four to 12 weeks. CT was performed every four to five weeks to evaluate the response. Both cases developed pseudoprogression, and 12 weeks was required to elicit a response to immunotherapy. Whenever disease progression was detected, we switched to second line chemotherapy as quickly as possible. Correct timing of changes in treatment is extremely important because of the characteristically rapid progression of CDC.

## 4. Conclusion

The present report discussed two cases of metastatic CDC in which PR and relatively long SD, respectively, were achieved by administering a combination of nivolumab and ipilimumab. To the best of our knowledge, the present report is the second to describe the treatment of CDC using a combination immunotherapy regimen. Establishing a standard treatment for metastatic CDC is crucial because of the poor prognosis of the disease. The findings of our study suggest that combination immunotherapy may be an effective treatment for metastatic CDC though further research is needed to verify the results.

## References

[1] Azuma T., Yao S., Zhu G., Flies A. S., Flies S. J., Chen L. (2008). B7-H1 is a ubiquitous antiapoptotic receptor on cancer cells. *Blood*.

[2] Dason S., Allard C., Sheridan-Jonah A. (2013). Management of renal collecting duct carcinoma: a systematic review and the Mcmaster experience. *Current Oncology*.

[3] Hodi F. S., O’Day S. J., McDermott D. F. (2010). Improved survival with ipilimumab in patients with metastatic melanoma. *New England Journal of Medicine*.

[4] Kuroda N., Tanaka A., Ohe C., Nagashima Y. (2013). Recent advances of immunohistochemistry for diagnosis of renal tumors. *Pathology International*.

[5] Koshkin V. S., Barata P. C., Zhang T. (2018). Clinical activity of nivolumab in patients with non-clear cell renal cell carcinoma. *Journal for ImmunoTherapy of Cancer*.

[6] Mizutani K., Horie K., Nagai S. (2017). Response to nivolumab in metastatic collecting duct carcinoma expressing Pd-L1: a case report. *Mol Clin Oncol*.

[7] Rimar K. J., Meeks J. J., Kuzel T. M. (2016). Anti-programmed death receptor 1 blockade induces clinical response in a patient with metastatic collecting duct carcinoma. *Clinical Genitourinary Cancer*.

[8] Watanabe K., Sugiyama T., Otsuka A., Miyake H. (2020). Complete response to combination therapy with nivolumab and ipilimumab for metastatic collecting duct carcinoma of the kidney. *Int Cancer Conf J*.

